# The Fate of Marine Bacterial Exopolysaccharide in Natural Marine Microbial Communities

**DOI:** 10.1371/journal.pone.0142690

**Published:** 2015-11-16

**Authors:** Zilian Zhang, Yi Chen, Rui Wang, Ruanhong Cai, Yingnan Fu, Nianzhi Jiao

**Affiliations:** State Key Laboratory of Marine Environmental Science, Institute of Marine Microbes and Ecospheres, Xiamen University, Xiamen, Fujian, People’s Republic of China; Texas A&M University at Galveston, UNITED STATES

## Abstract

Most marine bacteria produce exopolysaccharides (EPS), and bacterial EPS represent an important source of dissolved organic carbon in marine ecosystems. It was proposed that bacterial EPS rich in uronic acid is resistant to mineralization by microbes and thus has a long residence time in global oceans. To confirm this hypothesis, bacterial EPS rich in galacturonic acid was isolated from *Alteromonas* sp. JL2810. The EPS was used to amend natural seawater to investigate the bioavailability of this EPS by native populations, in the presence and absence of ammonium and phosphate amendment. The data indicated that the bacterial EPS could not be completely consumed during the cultivation period and that the bioavailability of EPS was not only determined by its intrinsic properties, but was also determined by other factors such as the availability of inorganic nutrients. During the experiment, the humic-like component of fluorescent dissolved organic matter (FDOM) was freshly produced. Bacterial community structure analysis indicated that the class Flavobacteria of the phylum Bacteroidetes was the major contributor for the utilization of EPS. This report is the first to indicate that Flavobacteria are a major contributor to bacterial EPS degradation. The fraction of EPS that could not be completely utilized and the FDOM (e.g., humic acid-like substances) produced *de novo* may be refractory and may contribute to the carbon storage in the oceans.

## Introduction

The oceans are the largest carbon reservoir on the planet and hence have significant impacts on global climate. An important form of marine storage of carbon is as organic carbon, predominantly dissolved organic carbon (DOC) [1]. It is known that more than 95% of DOC is refractory dissolved organic carbon (RDOC), which is resistant to microbial utilization and can be stored for several thousand years in the oceans [2]. Marine microbes have been proposed as major contributors to the generation of RDOC; however, the processes and mechanisms involved in the formation of RDOC remain unclear [3].

Chemical analysis of DOC in the ocean indicates that polysaccharides are one of the major components of seawater DOC, accounting for up to 50% of the DOC in surface waters and up to 25% of the DOC in deeper waters [4]. Among marine microbes, bacteria [5] and phytoplankton, such as diatoms [6], cyanobacteria [7] and dinoflagellates [8], are considered the major source of polysaccharides. Bacterioplankton represent the largest living surface in the world’s oceans and can exceed phytoplankton biomass even in the euphotic zone of oligotrophic regions [9]. It was indicated that capsular envelopes, which consist mainly of high-molecular-weight polysaccharides, are widely distributed in marine bacteria [5,10]. Now, numerous EPS producing bacteria have been isolated from marine environments, such as seawater, sediment, deep-sea hydrothermal vents, and sea ice [11]. EPS serves different functions in bacteria, such as the formation of a favorable microenvironment that facilitates attachment, maintenance of exoenzyme activity, sequestration of nutrients, and protection against toxins [12].

The composition of EPS varies considerably between phytoplankton and bacteria, which may in turn reflect its fate in the ocean. One of the major components of bacterial EPS is uronic acid, comprising up to 20–50% of the total polysaccharide fraction [13]. In contrast, phytoplankton EPS is relatively poor in uronic acid (< 5% of the total polysaccharide fraction) [13]. The negative charge associated with carboxyl groups of uronic acid of EPS has been implicated as a primary factor dictating complexation of these macromolecules with transition metals [13,14]. In turn, this property has been suggested to have a significant effect on the fate and ultimate degradation of EPS in the ocean [15]. As such, when compared to eukaryotic EPS, bacterial EPS is difficult to degrade and therefore tends to accumulate in marine environments [4,16]. However, factors influencing the bioavailability of bacterial EPS remain poorly understood.

The aim of this study was to clarify whether bacterial EPS rich in uronic acid is resistant to microbial utilization. To achieve this goal, we isolated EPS-producing bacteria from the South China Sea. The composition of EPS produced by JL2810, one of our marine bacterial isolates, was studied. EPS harvested from this organism was used to amend natural seawater, and the bioavailability of this EPS by native populations, in the presence and absence of ammonium and phosphate amendment, was investigated. Changes in the bacterial community structure during cultivation were also analyzed.

## Materials and Methods

### Bacterial isolation and cultivation

Seawater samples were collected from South China Sea during the cruise organized by National Natural Science Foundation of China on August 2012. Permit for the field study was obtained from National Natural Science Foundation of China. To isolate EPS-producing bacteria, aliquots (200 μL) of seawater were spread onto marine agar 2216E plates supplemented with 1.5% Agar. Morphologically different colonies were selected, inoculated into 4 mL of 2216E medium and incubated at 28°C for 3 days with agitation at 200 rpm, then supernatant was collected from the culture medium by centrifugation at 8,000 × g for 5 min. The content of the EPS in the supernatant was measured using the phenol/H_2_SO_4_ method with glucose (Glc) as the standard [17]. Among the isolates, bacterial strain JL2810, which was isolated from 75 m depth (115°E, 18°N), was found to produce large amount EPS and was selected as a representative strain for use in subsequence study.

The strain was maintained in a 2216E agar medium (MA; BD). A single colony of JL2810 was aseptically transferred to 2216E liquid medium and incubated at 28°C overnight with agitation at 180 rpm. After washing twice with 0.8% (w/v) NaCl, cells (20 mL) were inoculated into 2 L basal medium containing 1% Glc, 1.9% NaCl, 0.03% NH_4_Cl, 0.03% KCl, 0.04% K_2_HPO_4_, 0.05% MgSO_4_ ·7H_2_O, and 0.003% CaCl_2_ ·7H_2_O. The pH of the medium was 7.6. Cultivation was carried out at 25°C in a 3.7 L KLF 2000 bioreactor (Bioengineering, Wald, Switzerland) which was aerated at 1–1.5 vvm with constant agitation at 500 rpm. The pH was controlled at 7.6 using 0.25 M NaOH and 0.25 M sulfuric acid.

### EPS production

An aliquot (25 mL) of culture was collected each day from the bioreactor and 200 μL of this was used to measure the optical density (OD600) of the culture using a FlexStation 3 spectrophotometer (Molecular Device, USA). The remaining sample was filtered through a 0.22 μm Millex-GF filter unit and the supernatant was used to determine the concentration of Glc and EPS. Glc concentration was determined with a Lab Assay TM Glucose kit (Wako Pure Chemical Industries, Osaka, Japan). For quantification of EPS, three volumes of cold ethanol (-20°C) was added per volume of supernatant. The mixture was incubated overnight at 4°C and the precipitate was collected by centrifugation (12,000 × g, 30 min) and dissolved in deionized water.

### EPS isolation

At the end of cultivation, 500 ml of sample was collected and centrifuged at 8000 × g for 20 min with an AllegraTM64R (Beckman-Coulter, Fullerton, CA, USA). The supernatant was collected and filtrated through a 0.45 μm filter and then a 0.22 μm filter. Three volumes of cold ethanol (-20°C) was added to one volume of supernatant and the mixture was incubated at 4°C overnight. The precipitate was harvested by centrifugation (12,000 × g, 30 min), washed with 75% ethanol three times, and then dissolved in water. After centrifugation (12,000 × g, 30 min), the EPS solution was dialyzed (3500 Da cutoff) against water for 24 h. After dialysis, EPS was re-precipitated with 3 volumes of ethanol at 4°C overnight and then harvested by centrifugation as described above. The crude EPS isolated was freeze dried and stored in a desiccator at room temperature. Crude EPS was dissolved in water in the concentration of 5 g/L and then was purified using anion-exchange chromatography with a DEAE-Sepharose Fast Flow column (1.6 × 30 cm) (GE Healthcare). EPS was eluted with 20 mL of H_2_O at a flow rate of 0.5 mL/min followed by a NaCl gradient (30 mL) that ranged from 0 to 2 M at a flow rate of 1 mL/min. The process was carried out on an AKTA purifier FPLC system (Amersham Biosciences). Each elution fraction (2 ml) was collected and the EPS content was assayed using the phenol/sulfuric acid method. Major EPS-containing fractions were combined. After dialysis against distilled water, the EPS was collected and lyophilized.

### Analysis of EPS composition

Protein content of the EPS was quantified using the Coomassie brilliant blue G-250 protein assay [18]**.** Quantification of DNA was carried out using a PicoGreen DNA detection kit (QuantiFluorTM ds DNA, Promega). The C and N content was assayed with an elemental analyzer (PE 2400 II, Perkin Elmer).

The Molecular weight of the EPS was analyzed by high-performance liquid chromatography (HPLC). Separation was carried out on an Agilent 1260 Infinity HPLC system with refractive index detection via a TSK Gel GS3000SWXL column (7.8 × 300 mm) and a TSK Gel G2000SWXL column (7.8 × 300 mm), connected in series. Mobile phase was 0.1 M ammonium acetate containing 0.02% sodium azide solution. The column temperature was set at 30°C and flow rate was 0.6 mL/min. Standard solutions including dextran (Mw: 511, 167, 67, 40, 10, 5 and 1KDa) and maltotetraose (Mw: 666.6), and purified EPS sample were prepared at the concentration of 10 mg/mL in mobile phase and injected in duplicate. The injection volume was 10 μL.

Glycosyl composition analysis was performed by combined gas chromatography/mass spectrometry (GC/MS) of the per-O-trimethylsilyl (TMS) derivatives of the monosaccharide methyl glycosides produced from the sample by acidic methanolysis. Sample (300 μg) was taken and added to separate tubes with 20 μg of inositol as internal standard. Methyl glycosides were prepared from the dry sample following the mild acid treatment by methanolysis in 1 M HCl in methanol at 80°C (16 hours), followed by re-N-acetylation with pyridine and acetic anhydride in methanol (for detection of amino sugars). The sample was then per-O-trimethylsilylated by treatment with Tri-Sil (Pierce) at 80°C (0.5 hours). These procedures were carried out as described by Merkle and Poppe [19]. GC/MS analysis of the TMS methyl glycosides was performed on an Agilent 7890A GC interfaced to a 5975C MSD, using Agilent DB-1 fused silica capillary column (0.25 mm × 30m).

### Analysis of EPS utilization

Surface seawater was collected from the Xiamen coast (118°14´E, 24°29´N) in March, 2014. To analyze the potential utilization of EPS, the crude EPS (0.08 g/L) was added alone (treatment label: EPS) or in combination with inorganic nutrients (NH_4_Cl at a concentration of 26.7 mg/L and K_2_HPO_4_·3H_2_O at a concentration of 7.17 mg/L) (treatment label: EPS+N/P). For comparison, Glc (0.1 g/L) was also added alone (treatment label: Glc) or in combination with same amount of inorganic nutrients (treatment label: Glc + N/P). The C:N:P ratio was initially 106:16:1. Supplement of EPS (0.08 g/L), Glc (0.1 g/L) and same amount of inorganic nutrients (EPS+Glc+N/P) was also performed. Seawater without any amendment served as control. Each treatment was performed in two replicates. Two hundred mL of each treatment was performed in 500 mL Erlenmeyer flasks and these were incubated at 25°C with agitation at 160 rpm for 14 days. During cultivation, samples (4 ml) were taken at 12 h intervals for the first 5 days and at 24 h intervals during the rest of the cultivation period. The microbial growth (OD600), total sugar content, and Glc content of the samples were analyzed. OD600 and Glc content were measured as described above. Total sugar in the dissolved sample was measured using the phenol/H_2_SO_4_ method with Glc as the standard [17]. At the end of cultivation, samples (30 mL) were filtered (0.22 um, Milliex-GP Filter Unit) into Silicon boride bottles (pre-combusted, 450°C, 4 h) and stored at -20°C for fluorescent dissolved organic matter (FDOM) determination.

### Fluorescence measurement

Fluorescence measurement for FDOM was performed with fluorescence spectrophotometer (Cary Eclipse, Varian, USA) under a high sensitivity mode of 10 nm slit widths (excitation and emission) [20]. The Excitation Emission Matrix (EEM) measurement and normalization of fluorescence intensity were conducted according to methods described by Stedmon [21]. EEMs were generated by scanning emission spectra from 250 nm to 560 nm at 2 nm intervals, with 5 nm increments of the excitation wavelength from 200 nm to 450 nm. The EEM of Mill-Q water was subtracted from each sample and then EEMs were normalized to Raman units. The analysis was carried out in MATLAB with a “N-way toolbox for MATLAB” and split-half analysis which was used to validate the identified components.

### DNA extraction and PCR amplification

Microbial genomic DNA was extracted from 2 and 14 days’ cultures of each treatment using a PowerMax Soil DNA Isolation kit (MoBio Laboratories, Inc., Carlsbad, CA, USA) according to the manufacturer’s instructions. The resulting DNA (∼ 2 μg) from each sample was purified and concentrated via ethanol precipitation. DNA concentration was estimated using a NanoDrop 2000 spectrophotometer (Thermo Scientific, Waltham, MA, USA) and analyzed through gel electrophoresis. Q5 High-Fidelity DNA Polymerase (NEB) was used for the amplification of the V4 hypervariable region of 16S rRNA gene from microbial genome DNA using the universal primers (forward primer; 5’-AYTGGGYDTAAAGNG—3’ and reverse prime: 5’- TACNVGGGTATCTAATCC- 3’). The PCR reaction conditions were as follows: initial denaturation at 98°C for 5 min, followed by 27 cycles of 98°C for 30 s, 50°C for 30 s, and 72°C for 30 s, and extension at 72°C for 5 min. PCR product was excised from a 1.5% agarose gel and purified by AxyPrep DNA Gel Extraction Kit (Axygen Biosciences, Union, CA, USA).

### Illumina sequencing and 16S rDNA-based taxonomic analysis

The V4 amplicon was sequenced using pair-end method by Illumina Miseq with a 6 cycle index read. The average length of sequence reads was 221 bp. Sequence reads were assembled by Flash software (http://www.genomics.jhu.edu/software/FLASH/index.shtml) and the reads which could not be assembled were discarded. Only reads that had quality value (QV) scores of 20 for more than 99% of the sequence were extracted for further analysis. Chimera sequences were identified and removed using UCHIME. Sequences clustering was performed by UCLUST (QIIME) with a similarity cutoff of 97%, and clustered into operational taxonomic units (OTUs). The longest sequence in each cluster was chosen to be the representative sequences, which were annotated by RDP-classifier 2.2 (QIIME). The nucleotide sequences were submitted to GenBank under accession number SRP047314.

## Results

### Production of EPS by *Alteromonas* sp. JL2810

EPS-producing bacteria were isolated from seawater collected from the South China Sea. Among the isolates, strain JL2810, which produced the highest level of EPS, was selected for further use in this study. The 16S rRNA gene sequence of JL2810 exhibited 99.6% and 99.4% homology to that of *Alteromonas macleodii* ATCC27216T (CP003841) [22] and *Alteromonas marina* SW-47(T) (AF529060) [23], respectively. Therefore, JL2810 was identified as a strain of *Alteromonas*, and was designated *Alteromonas* sp. JL2810.

**Fig 1:**
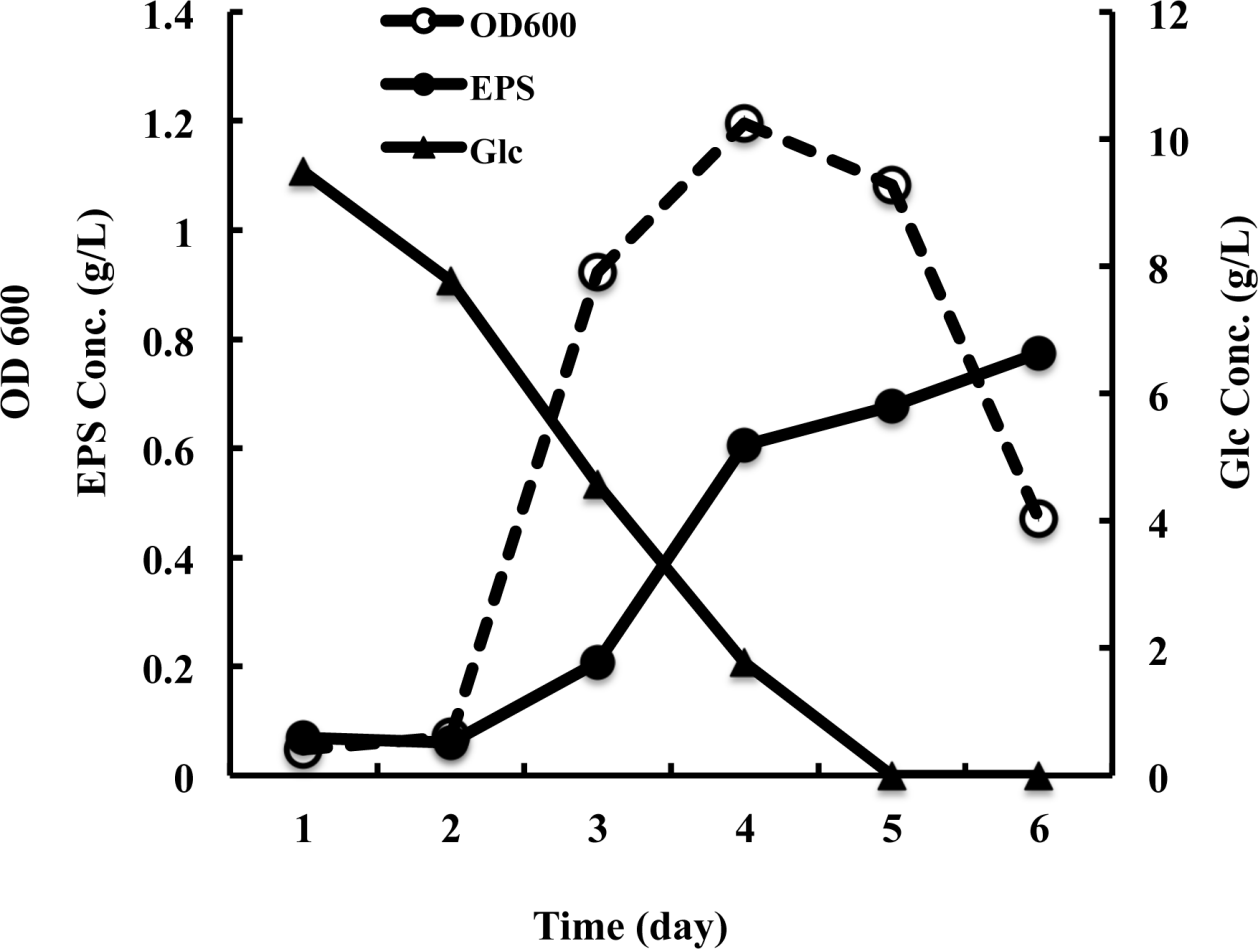
EPS production by JL2810. Time course of bacterial growth (OD600), EPS production, and Glc consumption during the cultivation of JL2810.

To analyze the EPS production, JL2810 was cultivated in a bioreactor at constant pH, dissolved oxygen and temperature. Glc was used as the sole carbon source. As shown in Fig 1, Glc was rapidly used by JL2810 cells, and complete utilization was observed by the fifth day. EPS was produced by JL2810 during the growth period and not reused by the cells at the late growth phase. At the end of cultivation, about 8% of Glc was converted to EPS, which reached a maximum of 0.77 g/L in the medium. Analysis of the EPS produced by JL2810 indicated it was primarily composed of carbohydrates, which accounted for 65% (w/w) of the EPS. Only a small amount of protein (0.92%) and DNA (1.08%) were detected. Elemental analysis revealed that C accounted for 33.84% of the total EPS mass, while N accounted for 0.88%. The C/N ratio of the EPS is about 38.

The molecular weight of the EPS is higher than 1.67 × 10^5^ Da, which is similar to the molecular weight of EPS (range 1–3 × 10^5^ Da) produced by most marine bacteria [11]. Analysis of the monosaccharide composition of the EPS indicated that the EPS contained a large amount of galacturonic acid (42.2%) and neutral monosaccharide including mannose (24.6%), rhamnose (16.2%), and Glc (14.4%). The high concentration of galacturonic acid in EPS produced by JL2810 is similar to that produced by other strains of *Alteromonas*, which were previously shown to contain 37–42% uronic acid [24].

### Utilization of EPS by native marine communities

**Fig 2:**
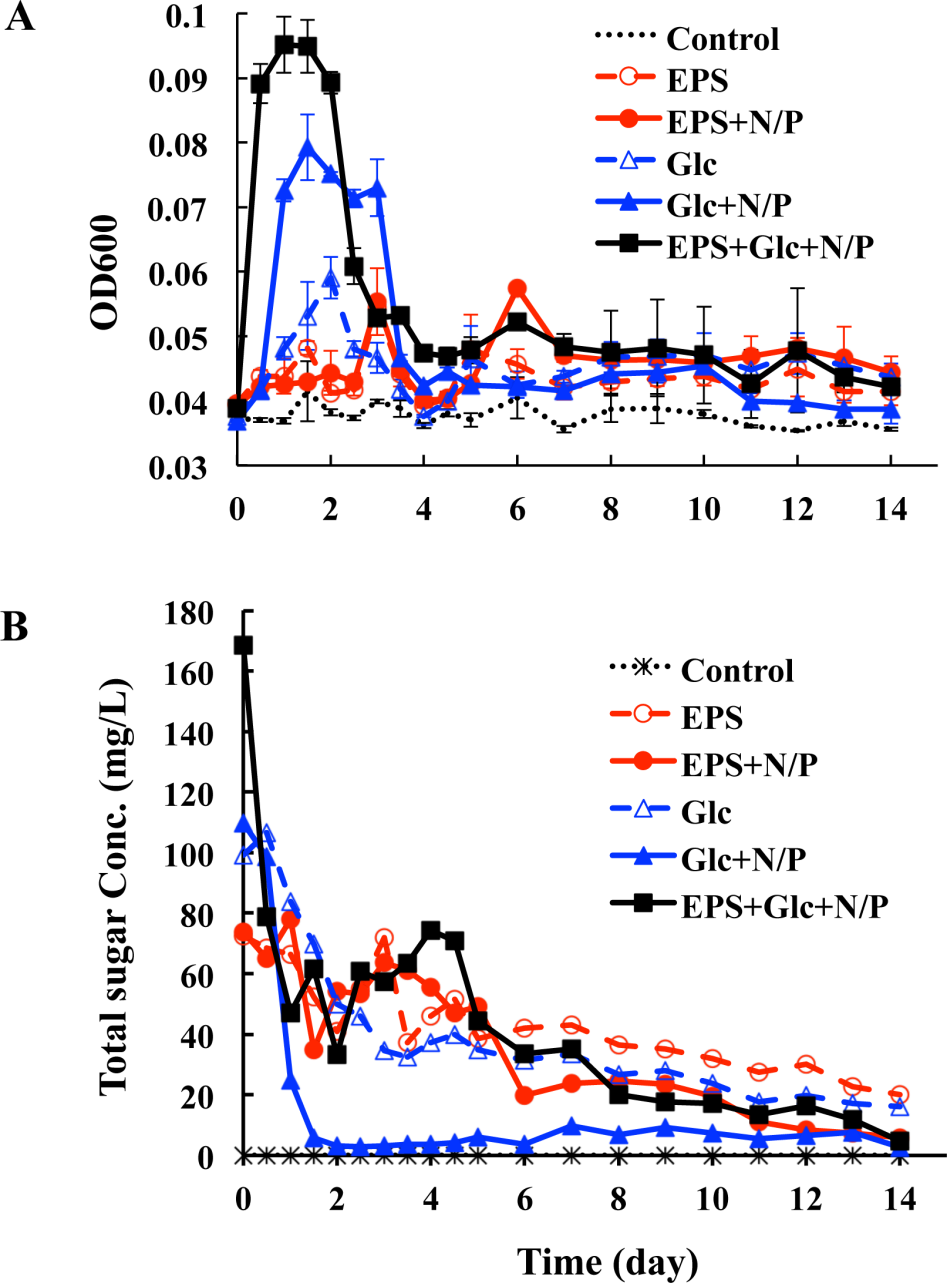
Effect of EPS and Glc on the microbial growth in seawater. (A) Growth curves. (B) Consumption of sugar during microbial cultivation.

To analyze the bioavailability of bacterial EPS, bioassay experiments were conducted. The EPS and Glc were added to natural seawater, either alone or in combination with inorganic nutrients of N and P, and the effect of these amendments on microbial growth was determined. As shown in the Fig 2A, no microbial growth was observed in unamend controls. In contrast, microbial growth (OD600) in seawater amended with Glc or Glc + N/P was about two times higher than that amended with EPS or EPS + N/P. Maximum growth was achieved in 1–2 d in seawater that was amended with Glc, whereas growth peaks were detected after 3 and 6 days of cultivation in seawater amended with EPS (Fig 2A). This result indicates that, when compared to Glc, the EPS was less bioavailable or was not as readily used for microbial growth.

Inorganic nutrient (N/P) amendment stimulated microbial growth, especially with Glc as the carbon source. A slight increase in microbial growth was also observed in EPS amended seawater with added N and P (EPS + N/P). As shown in the Fig 2A, microbial growth was nearly the same during the first four days of cultivation with EPS or EPS + N/P and was slightly increased by the addition of the inorganic nutrients N and P during later growth stages. Among the treatments examined, cell density in the EPS + Glc + N/P treatment was the highest.

### Consumption of Glc and EPS

Glc and total sugar concentrations were monitored during the cultivation period. As shown in Fig 2B, Glc was completely consumed within one day in the Glc + N/P treatment. When Glc alone was added, the consumption was slower, and ~20% of Glc remained at the end of the growth period. Total sugar concentrations in the two EPS treatments (EPS and EPS + N/P) decreased in a similar manner over the first five days of cultivation and were higher in the EPS treatment than in the EPS + N/P treatment from five to fourteen days (Fig 2B). At the end of cultivation, 27% of the added sugar remained in the EPS treatment, while only 8% remained in the EPS + N/P treatment. This result indicates that the consumption of both EPS and Glc are promoted by the addition of N and P.

### Production of FDOM

**Fig 3:**
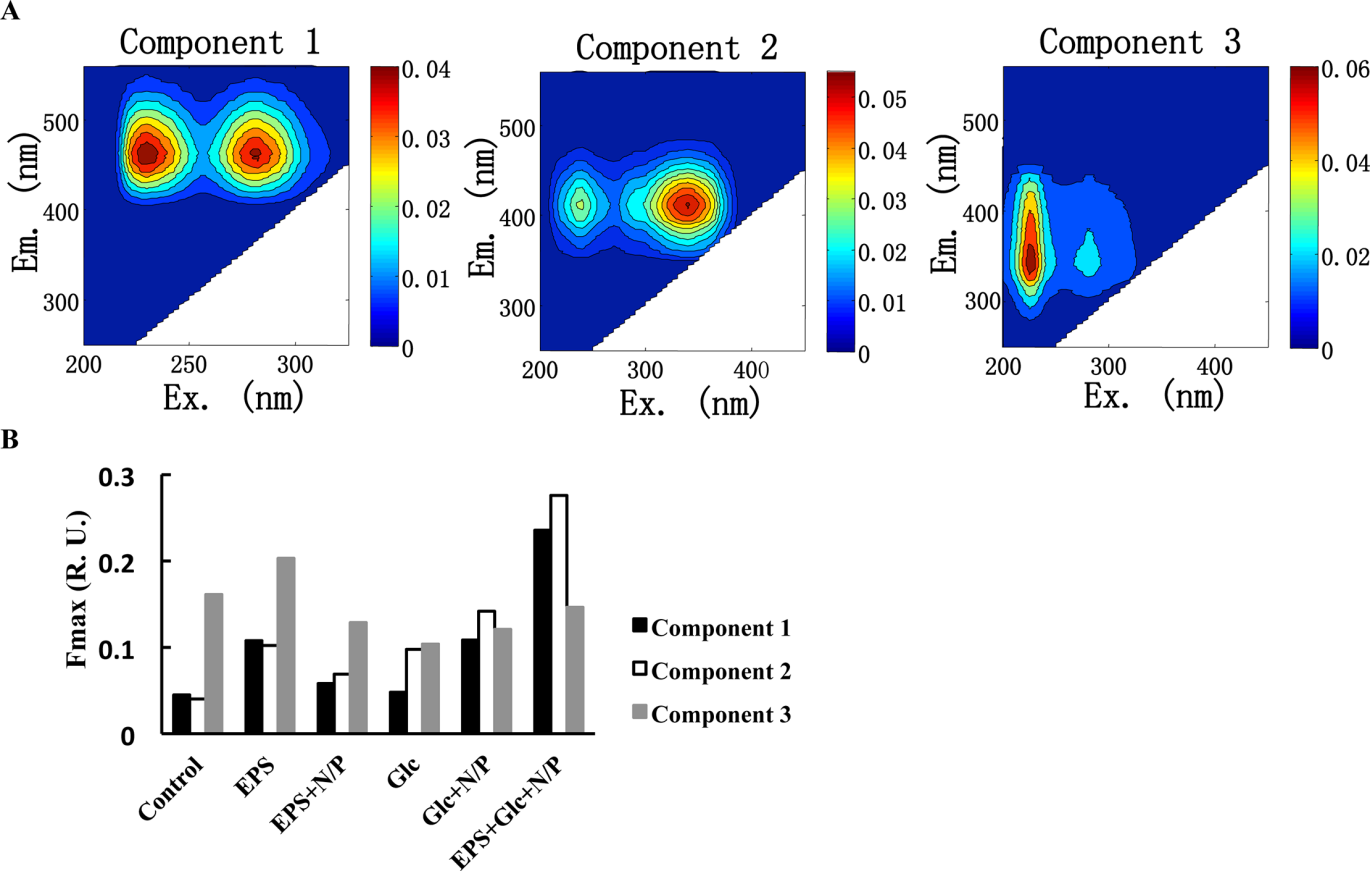
Three fluorescence components identified by the PARAFAC model. (A) Fluorescence graphs of the three components (component 1–3). (B) Fluorescence intensities of the three components after cultivation of seawater for 14 days.

Spectroscopic techniques provide a relatively simple method for tracking changes in the bulk chemical composition of DOM. In particular, the optically active fraction of DOM that fluoresces is inherently related to the chemical and structural composition of DOM [25]. The interpretation of EEM derived from fluorescence spectroscopy has been developed substantially with the aid of a powerful multivariate statistical approach called parallel factor (PARAFAC) analysis [26]. According to PARAFAC modeling analysis, three FDOM components can be detected in the culture medium at the end of the incubation: two humic-like components (components 1 and 2) and one protein-like component (component 3) (Fig 3A). The chemical properties of component 1 (excitation/emission wavelength (Ex/Em) of 312 nm/380-420 nm) are similar to those of terrestrial humic-like components. Likewise, component 2 (Ex/Em of 340 nm/440 nm) was similar to marine humic-like components. The fluorescence of component 3 (Ex/Em of 280 nm/350-420 nm) was similar to a protein-like component [27]. Compared with the control treatment, the fluorescence intensities of components 1 and 2 in Glc and Glc + N/P groups were greater (Fig 3B). Fluorescence intensities of the two humic-like components in the EPS group were relative high and were reduced when seawater was amended with N/P. The fluorescence intensities of components 1 and 2 were the highest in the EPS + Glc + N/P treatment, and the values were nearly twice as intense as the individual EPS and Glc treatments. Except for the EPS treatment, the fluorescence intensity of component 3, a protein-like component, was low in all treatments when compared to the control. These results indicated that components 1 and 2 (humic acid-like) are highly produced during cultivation when compared to component 3 (protein-like).

### Changes in bacterial community structure

To understand the microbial community involved in the utilization of EPS and Glc, the bacterial community structures in the 2-day (d) and 14-d incubations of each treatment were analyzed. The rarefaction curves obtained for the V4 region of 16S rRNA gene sequences nearly reached saturation for many samples (S1 Fig). After the quality control, a total of 588,448 validated sequence reads were obtained, which were classified into a total of 6,546 different OTUs based on an identity level of 97%. The coverage ranged from 97.8–98.7%, suggesting that the number of sequences in each samples was enough to reflect the real bacterial diversity. Of these OTUs, 5486 and 4606 OTUs were classified as phylum and class levels, respectively.

**Fig 4:**
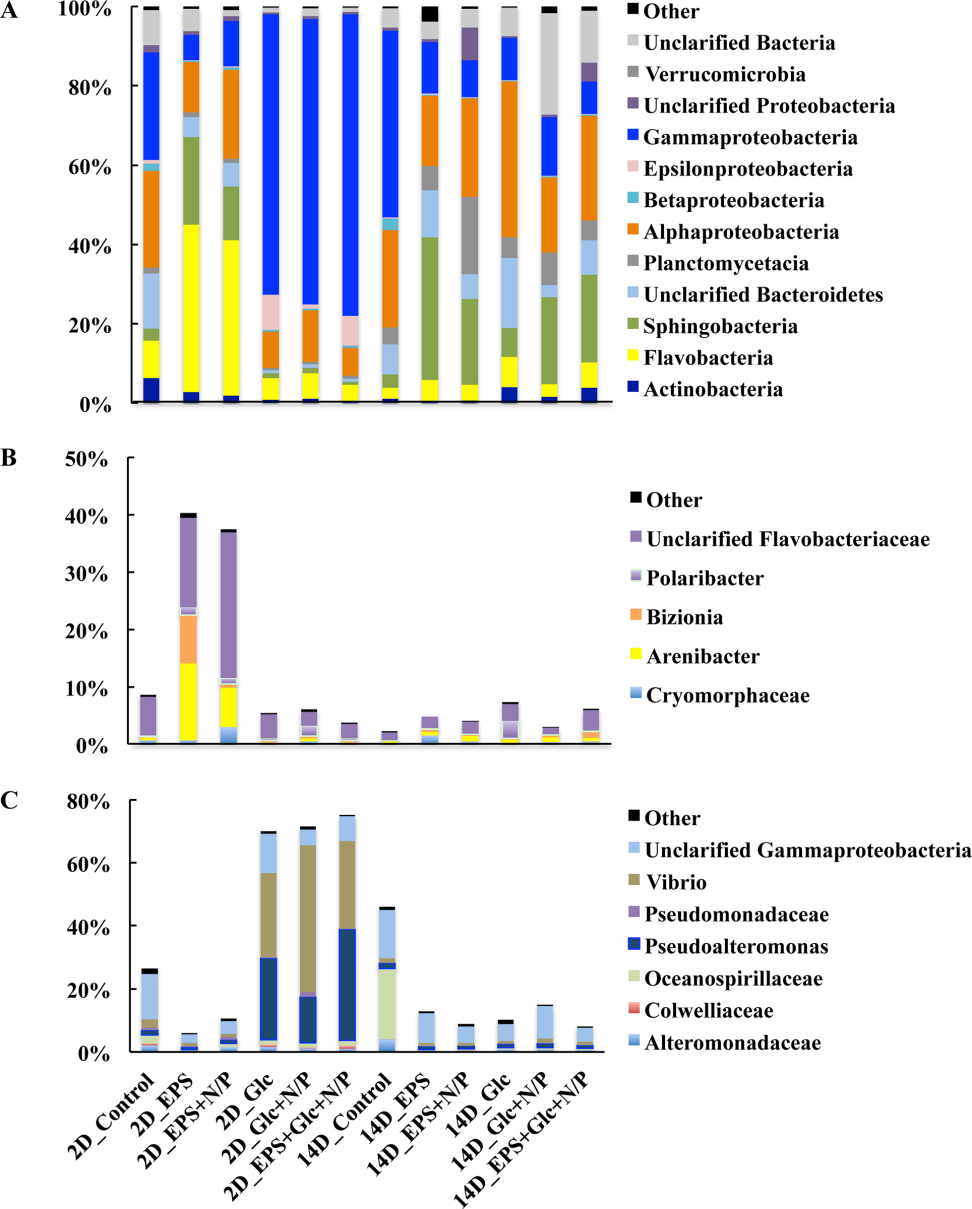
Bacterial community composition analysis. Phylogenetic compositions of the bacteria in the 2-d (2D) and 14-d (14D) cultures were analyzed. The relative abundance of each class is expressed as the mean percentage of total sequences obtained in the treatment. The major classes (A) and genus belonging to Flavobacteria class (B) and Gammaproteobacteria class (C) were indicated.

We classified the OTUs based on their phylogenetic affiliations and determined their relative proportions in the total reads. In 2-d cultures, the phylogenetic affiliation of microbes in the control was assigned to 21 bacterial and one archaeal phyla, with the dominant phyla being Proteobacteria (55.1%), Bacteroidetes (Cytophaga-Flavobacteria-Bacteriodes) (26.0%), Actinobacteria (6.1%), Verrucomicrobia (1.8%) and Planctomycetes (1.5%) (S2 Fig). The minor phyla (<1% of total microbial sequences) (Other, S2 Fig) were Euryarchaeota, Acidobacteria, Aquificae, Bacteria_incertae_sedis, Chlamydiae, Chloroflexi, Cyanobacteria, Deinococcus-Thermus, Firmicutes, Fusobacteria, Gemmatimonadetes, Lentisphaerae, Nitrospira, OD1, Spirochaetes, Tenericutes, and WS3. The bacterial community composition changed dramatically from Proteobacteria toward Bacteroidetes in the 2-d cultures supplemented with EPS (S2 Fig). Bacteroidetes accounts for 67% (EPS) and 56% (EPS + N/P) of the total microbial sequences (S2 Fig). Flavobacteria was identified as the major contributor of the Bacteroidetes response to EPS treatment, followed by Sphingobacteria. The Flavobacteria accounts for 41% and 38% of total microbial sequences in EPS and EPS + N/P, while Sphingobacteria accounts for 21% and 13% (Fig 4A), respectively. In the three Glc supplemented cultures, on the other hand, the Proteobacteria phylum was the predominant microbial group, with members of the four major classes of Alpha-, Beta-, Gamma-, and Epsilonproteobacteria accounting for 87–92% of the total microbial sequences (Fig 4A). Gammaproteobacteria, accounting for 70–76% of the total microbial sequences, was identified as the predominant class of Proteobacteria in the Glc, Glc + N/P, and EPS + Glc + N/P cultures (Fig 4A). Compared to the EPS and Glc alone treatments, the Alphaproteobacteria and Gammaproteobacteria classes were relatively abundant when inorganic nutrients (N and P) were supplemented. Other groups were rare, and many sequences were present at abundance < 1% of the total population (Other, Fig 4).

In the 14-d cultures, Bacteroidetes (53%) was the predominant microbial group only in the culture sample supplemented with EPS, while Proteobacteria (35%-74%) was the predominant microbial group in other samples (Fig 4A). The relative abundance of Flavobacteria dramatically decreased in the 14-d cultures (4.5%, on average) amended with EPS (EPS and EPS + N/P) than that in 2-d cultures (40%, on average). Gammaproteobacteria also decreased in the seawater supplemented with Glc (Glc, Glc + N/P and EPS + Glc + N/P) from about 70% (in 2-d cultures, on average) to 1% (in 14-d cultures, on average). At the end of cultivation, the relative abundance of Sphingobacteria of Bacteroidetes was relatively higher than in the control. The data indicated that the dynamic change in the bacterial community structure responded to the carbon source present during cultivation; also, the members of Flavobacteria are important for the utilization of EPS, while those of Gammaproteobacteria are important for the utilization of Glc.

Due to the overwhelming sequence abundance, the composition of the classes of Flavobacteria and Gammaproteobacteria were scrutinized in more detail. Two families of Flavobacteriaceae and Cryomorphaceae, belonging to Flavobacteria, were identified in the cultures, with Flavobacteriaceae as the predominant group (Fig 4B). The genus *Arenibacter* in the family Flavobacteriaceae specifically responded to EPS treatment. The unclarified Flavobacteriaceae accounts for 15% (EPS) and 25% (EPS + N/P) of the sequences in the 2-d cultures (Fig 4B). Two genera *Pseudoalteromonas* and *Vibrio*, belonging to Gammaproteobacteria, were predominant in the Glc supplemented cultures (Fig 4C), indicating that growth of the members of these genera were specifically stimulated by Glc.

## Discussion

Heterotrophic microbial communities are responsible for re-mineralizing and transforming a considerable fraction of autochthonous marine DOC [28] that comprise most of the organic matter in marine systems [29]. These communities are active components in the marine pelagic food web, leading to an efficient cycling of carbon and energy in the microbial loop [30]. They convert labile DOC into biomass, CO_2_, and RDOC that can be stored for long periods in global oceans. Members of the genus *Alteromonas* are increasingly recognized as an active component of marine communities. They are globally distributed in the ocean and have a copiotrophic way of life, and as γ-strategists, they can grow rapidly when fresh carbon is released into marine habitats [31,32]. Strains of *Alteromonas* typically produce EPS [33,34] and are free living or associated with particles [31,35]. In the South China Sea, *Alteromonas* was isolated from seawater samples from all depths and identified as a dominant taxon (data not shown).

The EPS producing strain JL2810 was selected for this study. The strain converted about 8% Glc to EPS within five days of cultivation. The EPS produced by JL2810 had a molecular weight > 1.67 × 10^5^ Da and contained large amount (42.2%) of galacturonic acid as uronic acid sugar, which is similar to that produced by most marine bacteria. Therefore, the EPS used in this study is likely a representative of bacterially produced EPS in marine systems.

Our data indicated that EPS is less efficient for microbial growth than the monosaccharide Glc, suggesting that the bioavailability of carbohydrates depends partly on their chemical composition. Previous studies suggested that only small molecules, less than ~600 Da [36], can be transported across the cellular membrane. There is evidence that the incorporation rate of large molecules of bacterial capsular material from bacterioplankton is lower than the corresponding rates for Glc uptake [37]. Therefore, the high molecular weight EPS must be hydrolyzed to small molecular organic matter prior to its transport and use inside the cell; hydrolysis is a recognized rate-limiting step in the microbial utilization of DOM [38]. The enzymes for hydrolysis of EPS are usually highly specific, and their specificity is determined by carbohydrate structure, with uronic acids often playing a major role [39]. The EPS produced by JL2810 is a heteropolysaccharide containing mannose, rhamnose, Glc, galactose, and a relatively large amount of galacturonic acid. Thus, the hydrolysis of this EPS might require a diverse set of enzymes [39].

The EPS could not be completely utilized during the 14 d cultivation experiments, suggesting that the remaining EPS molecules might be difficult for microbes to utilize. To confirm this hypothesis, a longer incubation would need to be used. The composition and molecular features of the remaining EPS molecules also need to be analyzed. Consumption of Glc was largely promoted by inorganic nutrients (Glc+N/P). On the other hand, the consumption of EPS only was promoted at the late growth stages (Fig 2B), suggesting that EPS may contain N and P itself; so, the N and P are not the limiting factors during the initial utilization of EPS but are likely to become limiting during later stages of breakdown. Similarly, it was indicated that N and P contained in algal organic material was important for bacterial growth and the utilization of the organic material [40]. In support of this hypothesis, a small amount of protein and DNA was detected in the EPS, which may provide N and P for microbial growth. It may also suggest that there is a lag time before a natural population can utilize EPS or the microbes that can use EPS take longer to grow and become more abundant later in the experiment. Without nutrient supplementation, both Glc and EPS cannot be completely utilized by the microbial assemblages. These results indicate that the bioavailability of DOC is not only determined by its composition but is also affected by inorganic nutrient availability. When inorganic nutrients are replete, DOC can be mobilized for degradation [41]. The increased microbial growth in the presence of inorganic N and P might account for the increased Glc and EPS consumption. EPS production usually increases when marine bacteria are grown in laboratory conditions with a limited availability of nutrients, such as nitrogen and phosphorus [42]. Taken together, these data suggest that inorganic nutrient limitation may shift DOC towards longer residence times in the ocean.

Consumption of Glc and EPS was linked to the production of humic-like components 1 and 2 (Fig 3). As shown in Fig 4, Glc and EPS were utilized by different bacterial community, suggesting that different humic acid-like components might be produced in Glc and EPS treatment incubations. Component 1 is often used as a tracer for terrestrial CDOM in coastal environments, but it can originate from either terrestrial or marine microbial sources [43]. Component 2 has been widely observed in the seawaters and is hypothesized to originate from autochthonous processes [44]. Production of component 2 by bacteria was also indicated by Romera-Castillo [45]. Humic-like components have been traditionally considered refractory components that resist bacterial degradation and can be accumulated in the ocean on centennial to millennial time scales [46,47]. The protein-like component 3 decreased in fluorescence intensity during cultivation in all treatments, with the exception of EPS treatment. Relatively higher intensities of component 3 detected in the EPS group may partly originate from the EPS itself; the addition of N and P promoted EPS degradation and reduced the fluorescence of this component (Fig 3). This hypothesis was supported by the identification of protein-like fluorescence substance with Ex/Em similar to the component 3 in extracellular polymeric substances [48]. Analysis of the distribution of FDOM in the ocean revealed that the fluorescence intensity levels of the protein-like component were highest in the surface waters and decreased with depth, but did not change systematically in the bathypelagic layer (1000 m-bottom). However humic-like components were lowest in the surface and increased with depth in the mesopelagic layer (200–1000 m), and then slightly decreased with depth in the bathypelagic layer [20,43]. These result suggest that component 3 is more bioavailable and is more readily used than humic-like components [43]. Thus, the formation of humic-like components during the consumption of bacterial EPS might contribute to the RDOC pool in the ocean.

Microbial community structure analysis indicated that Proteobacteria and Bacteroidetes are the major phyla involved in the consumption of monosaccharide Glc and bacterial EPS, respectively (S2 Fig). It is known that Proteobacteria and Bacteroidetes are the most abundant heterotrophic bacteria in the ocean [49]. The bacterial community composition changed dramatically from Proteobacteria towards Bacteroidetes after 2 d cultivation with EPS as carbon source (S2 Fig). In fact, representatives of Bacteroidetes have been increasingly recognized as specialists for the degradation of macromolecules. For example, it has been indicated that the Flavobacteria and Sphingobacteria of Bacteroidetes often appear in oil-polluted seawater, indicating their ability to degrade relatively refractory hydrocarbons [50]. The relative abundance of Flavobacteria was about 40% in the community structure of the 2-d culture supplemented with EPS, but decreased to about 5% in the 14-d culture. The result suggested that the Flavobacteria specifically responded to the EPS treatment and that they may be involved in the hydrolysis of EPS. It is known that the members of Flavobacteria are especially proficient in degrading various biopolymers, such as cellulose, chitin, and pectin [51] and thus make a significant contribution to the remineralization of marine organic matter [49]. In addition, Flavobacteria isolated from lake water decompose refractory substrates more efficiently than do other strains such as *Pseudomonas* and *Erkinia* belonging to the phylum Proteobacteria [52]. Genomic analysis indicated that the marine flavobacterium *Formosa agariphila* KMM 3901T has the capacity to degrade a wide range of algal polysaccharides [53]. Further growth experiments confirmed that the strain can use abundant algal polysaccharides [53]. Enzymes involved in the degradation of polysaccharides from the cell walls of green algae rich in uronic acid were also identified from marine bacteria of Flavobacteria [54]. In our study, the genus *Arenibacter*, belonging to the class Flavobacteria, was abundant in the cultures supplemented with EPS (Fig 4B). Recently, it was indicated that the strains of the genus *Arenibacter* can synthesize a wide spectrum of glycosidases, especially β-N-acetylglucosaminidases and α-N-acetylgalactosaminidases, and may participate in the degradation of natural polysaccharides in marine environments [55]. The EPS used in this study was isolated from marine bacterium JL2810 and is rich in galacturonic acid; therefore, the enzymes synthesized by members of *Arenibacter* might be important for the utilization of the EPS. The growth of genus *Bizionia* was specifically promoted in the 2-d culture amended with EPS (Fig 4B). The genus *Bizionia* was first described by Nedashkovskaya et al. [56], and since then, few species have been isolated. The utilization of EPS by these species has not been described. The role of the members of *Bizionia* in the ocean carbon cycle is largely unknown. The mechanism of growth of *Bizionia* in the culture supplemented with EPS without N/P need to be further addressed. In this study, we only analyzed the bacterial community composition in 2- and 14-d cultures, since maximum bacterial growth was detected in 2-d culture in preliminary experiment amended seawater with Glc. However, we noticed that growth peaks were also detected after 3 and 6 days of cultivation in seawater amended with EPS, suggesting that bacterial community composition may changed significantly during the cultivation in EPS treatment groups.

When Glc served as the carbon source, the Gammaproteobacteria class was the dominant bacteria, suggesting that the members of Gammaproteobacteria are more sensitive to the labile DOC such as Glc. Two genera *Pseudoalteromonas* and *Vibrio* belonging to the class Gammaproteobacteria were predominant in the Glc-supplemented cultures (Fig 4C). This result is consistent with the previous observations that the copiotrophic organisms, often belonging to clades within the Gammaproteobacteria (e.g. *Vibrio*, *Alteromonas*, *Pseudoalteromonas*), could rapidly reach numerical dominance within labile DOM (e.g. Glc, gluconic acid) supplemented treatments [57] and are considered opportunistic r-strategists because of their short generation times and rapid growth response [58].

## Conclusions

This study showed that bacterial EPS rich in uronic acid is less efficient for microbial utilization than the monosaccharide Glc. Consumption of EPS and Glc was promoted by the inorganic nutrients N and P. Flavobacteria and Gammaproteobacteria are the major contributors to the utilization of EPS and Glc, respectively. The humic-like components synthesized *de novo* and the fraction of EPS that cannot be hydrolyzed might contribute to the formation of RDOC in the ocean. The structure and composition of the EPS that remained in the culture system need to be further analyzed in the future.

## Supporting Information

S1 Fig Diversity of microbial communities. The total numbers of screened clones are plotted against the unique operational taxonomic units (OTUs) in the 2-d (2D) and 14-d (14D) culture samples.
(PPTX)

S2 Fig Bacterial community composition analysis at phylum level. (PPTX)
