# Supplementary figures and images for: The Fate of Marine Bacterial Exopolysaccharide in Natural Marine Microbial Communities
Source: PLoS One. 2015 Nov 16;10(11):e0142690. doi: 10.1371/journal.pone.0142690 (PMC4646686; doi:10.1371/journal.pone.0142690)

## Slide 1
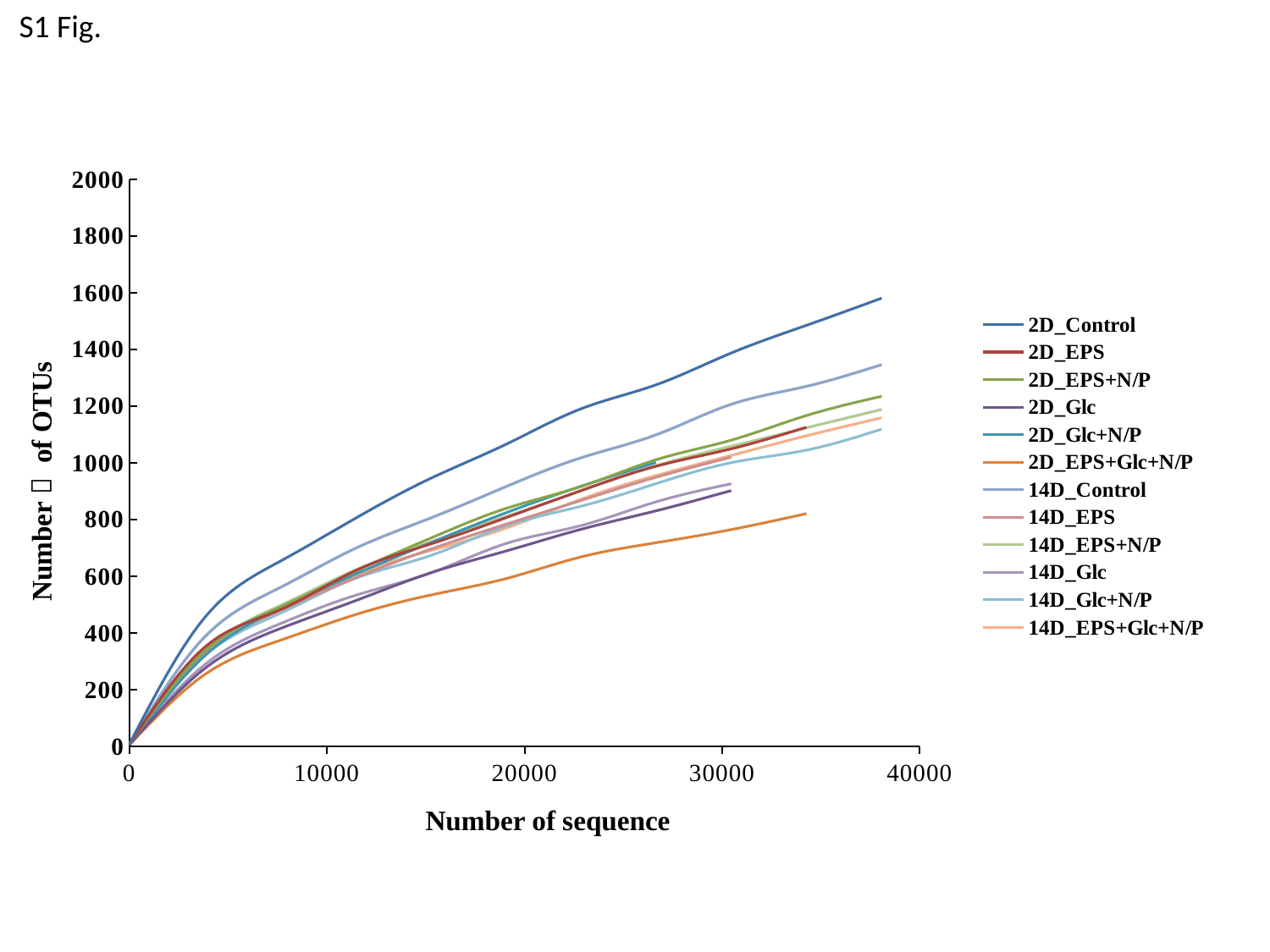

S1 Fig.
### Chart
| Category | | | | | | | | | | | | |
|---|---|---|---|---|---|---|---|---|---|---|---|---|Numberｒ of OTUs
Number of sequence

Supplement: S1 Fig — The total numbers of screened clones are plotted against the unique operational taxonomic units (OTUs) in the 2-d (2D) and 14-d (14D) culture samples. (PPTX) [file pone.0142690.s001.pptx]
