# Supplementary material for: The Fate of Marine Bacterial Exopolysaccharide in Natural Marine Microbial Communities
Source: PLoS One. 2015 Nov 16;10(11):e0142690. doi: 10.1371/journal.pone.0142690 (PMC4646686; doi:10.1371/journal.pone.0142690)
Supplement: S2 Fig — (PPTX) [file pone.0142690.s002.pptx]

## Slide 1
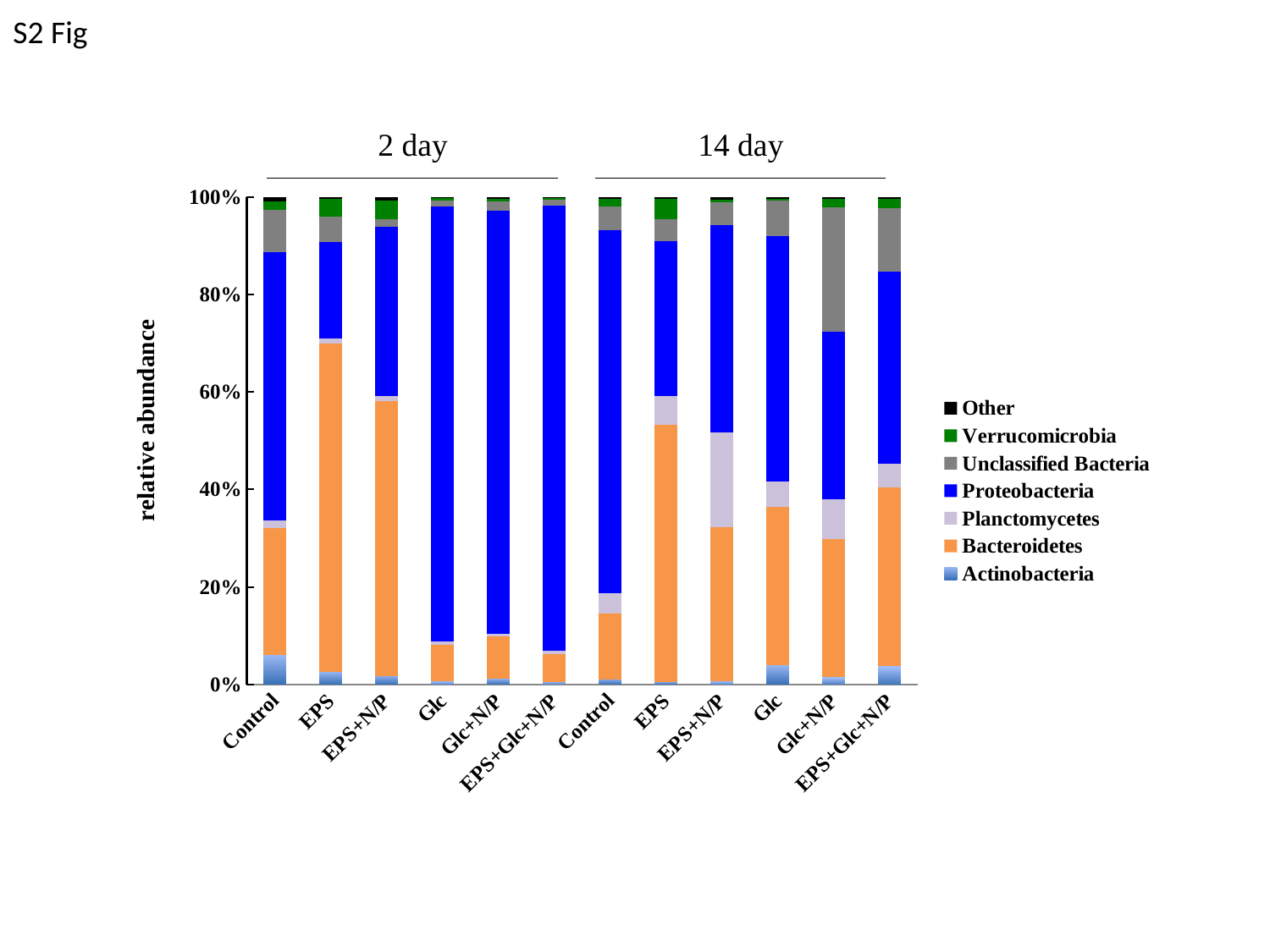

S2 Fig
2 day
14 day
### Chart
| Category | Actinobacteria | Bacteroidetes | Planctomycetes | Proteobacteria | Unclassified Bacteria | Verrucomicrobia | Other |
|---|---|---|---|---|---|---|---|
| Control | 6.093145725339999 | 25.9586110425 | 1.53460051016 | 55.1016209989 | 8.61721385666 | 1.81848103349 | 0.87632683288012 |
| EPS | 2.57009973923 | 67.2661773799 | 1.18557948222 | 19.6574992607 | 5.352582197489997 | 3.5540500578 | 0.41401188267823 |
| EPS+N/P | 1.73209575979 | 56.37046053969997 | 1.13866910124 | 34.5709068777 | 1.54189490769 | 3.96885778048 | 0.67711503347591 |
| Glc | 0.689347035169 | 7.48388332163 | 0.5776472841 | 89.2385268399 | 1.30848279824 | 0.465947533031 | 0.23616518797436 |
| Glc+N/P | 1.09006493288 | 8.70369747334 | 0.629142414965 | 86.76782289809998 | 1.96817279548 | 0.484473303502 | 0.35662618174476 |
| EPS+Glc+N/P | 0.539275003946 | 5.653180407219995 | 0.670805492713 | 91.2742673752 | 1.23375598464 | 0.476140369338 | 0.15257536697011 |
| Control | 1.07817897771 | 13.5124430655 | 4.057473540609997 | 74.4713622461 | 4.83200211235 | 1.60186590974 | 0.44667414790913 |
| EPS | 0.513080571548 | 52.6534021418 | 6.03466276885 | 31.7453688512 | 4.40294723026 | 4.23888076843 | 0.41165766787033 |
| EPS+N/P | 0.600388023701 | 31.6422840963 | 19.4247810812 | 42.5960883016 | 4.70347648262 | 0.448324681454 | 0.58465733312373 |
| Glc | 3.890641430069997 | 32.57389882 | 5.08236943568 | 50.4498189041 | 7.24967870078 | 0.441056198154 | 0.31253651127429 |
| Glc+N/P | 1.51955019844 | 28.2283551374 | 8.130604145229999 | 34.5196971924 | 25.48324268699997 | 1.78597677495 | 0.33257386447168 |
| EPS+Glc+N/P | 3.75839410788 | 36.6235829302 | 4.90649144343 | 39.35482706329994 | 13.0334320168 | 1.85392447108 | 0.46934796736236 |relative abundance
